# Smooth Muscle Cell Alignment and Phenotype Control by Melt Spun Polycaprolactone Fibers for Seeding of Tissue Engineered Blood Vessels

**DOI:** 10.1155/2015/434876

**Published:** 2015-09-01

**Authors:** Animesh Agrawal, Bae Hoon Lee, Scott A. Irvine, Jia An, Ramya Bhuthalingam, Vaishali Singh, Kok Yao Low, Chee Kai Chua, Subbu S. Venkatraman

**Affiliations:** ^1^Materials and Science Engineering, Nanyang Technological University, N4.1-01-30, 50 Nanyang Avenue, Singapore 639798; ^2^School of Mechanical & Aerospace Engineering, Nanyang Technological University, 50 Nanyang Avenue, Singapore 639798; ^3^Department of Polymer and Process Engineering, Indian Institute of Technology Roorkee, Roorkee, Uttarakhand 247667, India; ^4^School of Biological Sciences, Nanyang Technological University, 60 Nanyang Drive, Singapore 637551

## Abstract

A method has been developed to induce and retain a contractile phenotype for vascular smooth muscle cells, as the first step towards the development of a biomimetic blood vessel construct with minimal compliance mismatch. Melt spun PCL fibers were deposited on a mandrel to form aligned fibers of 10 *μ*m in diameter. The fibers were bonded into aligned arrangement through dip coating in chitosan solution. This formed a surface of parallel grooves, 10 *μ*m deep by 10 *μ*m across, presenting a surface layer of chitosan to promote cell surface interactions. The aligned fiber surface was used to culture cells present in the vascular wall, in particular fibroblasts and smooth muscle cells. This topography induced “surface guidance” over the orientation of the cells, which adopted an elongated spindle-like morphology, whereas cells on the unpatterned control surface did not show such orientation, assuming more rhomboid shapes. The preservation of VSMC contractile phenotype on the aligned scaffold was demonstrated by the retention of *α*-SMA expression after several days of culture. The effect was assessed on a prototype vascular graft prosthesis fabricated from polylactide caprolactone; VSMCs aligned longitudinally along a fiberless tube, whereas, for the aligned fiber coated tubes, the VSMCs aligned in the required circumferential orientation.

## 1. Introduction

As research in implantable biomaterial advances, the understanding and manipulation of cell-substrate interactions have increased in importance. One approach is to produce a more biomimetic construct that can recruit and control the patterning of functional cells to mimic the native tissue organization. For example, the aligned orientation of cells on extracellular matrix (ECM) plays an important role in several tissues including corneal stroma, tendons, bones, skeletal muscle, and, with significance to the present study, the vasculature [1]. Here we demonstrate an intrinsically effective cell aligning surface fabricated from the biodegradable and cytocompatible polymers PCL, chitosan, and gelatin [2].

The development of a small diameter vascular prosthesis (>6 mm diameter) for arterial disease has been hampered by the mechanical compliance mismatch of the prosthesis and the native blood vessel. The mismatch is the key factor for the relatively rapid loss of patency compared to larger-diameter prostheses. In turn the mismatch is due to the fact that artificial prostheses do not mimic the layered structure of the native vessel, in which one of the layers has circumferentially aligned vascular smooth muscle cells (VSMCs) as well as extracellular matrix (ECM) [3].

The two predominant cell types within blood vessels, fibroblasts and VSMCs, could both functionally benefit cell-seeded prosthesis if recruited in an aligned orientation [1, 4]. Fibroblasts produce extracellular matrix such as collagen fibrils and elastin [5] that confer to blood vessels most of their mechanical and structural properties. In the context of a vascular prosthesis it is beneficial to align the growth of cells to control the pattern of ECM deposition. If the scaffold of the prosthesis is biodegradable, then the construct will be eventually replaced by ECM with the desired orientation [6].

Of particular interest to this study is the alignment of VSMCs. These cells are integral to the vascular functioning through regulation of vessel tone and lumen diameter. Interestingly, these cells exist as two very distinct and changeable phenotypes: the contractile, characterized by a spindle shape and the abundant presence of alpha-smooth muscle actin (*α*-SMA), and the secretory (also referred to as synthetic), recognizable by rhomboid shape and reduced *α*-SMA [7]. The contractile VSMC allows the changes that mediate blood pressure, by altering the vessel luminal diameter, and is not proliferative, whereas the secretory phenotype is associated with tissue remodeling, inflammation, and proliferation. The secretory phenotype is central to the pathology of neointimal hyperplasia and artery bypass failure. There is plasticity between the two states as they are not differentiation end-points [7, 8].

During the culturing of VSMC, freshly seeded VSMCs exist primarily in the contractile state, but over time the population shifts predominantly towards the secretory phenotype. For long-term patency, it is important to preserve the contractile phenotype, as only VSMCs in the contractile state are beneficial in fabricating cellularized tissue engineered blood vessel (TEBV) because this phenotype minimizes the compliance mismatch. Furthermore, the cells must be circumferentially orientated to direct their function [9].

Previous studies already have demonstrated that the aligned orientation and phenotypic characteristics of cells can be guided by surface cues generated through micropatterning a surface with channels [10]. These channels are often considerable wider and deeper than the dimensions of the cell, for example, 60 *μ*m deep and 300 *μ*m wide [9]. Although these scaffolds encourage cell alignment the channel depth often prevents cell interaction across the interval. Cells are able to align on grooves as shallow as 150 nm [11]; however deeper channels are more effective for cell alignment, down to depths of approximately 25 *μ*m [12], whereas, for groove width, as it increases cellular alignment decreases as cells lying centrally can longer sense the edges [13, 14].

Here, we construct a scaffold of parallel melt spun PLC fibers. As the fiber width is 10 *μ*m, a scaffold of side-by-side fibers can be formed to orientate the cells within a groove of 10 *μ*m width with no overly large groove walls nor any extended interval between grooves, thus enabling intercellular contact.

Early alignment studies employed nondegradable polymers such as channels on PDMS films to produce aligned cell-orientating scaffolds. More recently, however, biodegradable polymers have been studied, since such scaffolds can be slowly replaced by native tissue and ECM which minimizes the issue of thrombosis. Such polymers have included P[(LLA-CL)] [15], PCLLGA [9]. Here we use the biodegradable polymers polycaprolactone and polylactide caprolactone. These polymers have been promoted as suitable for fabricating a vascular conduit and such an application is actively investigated by several groups [16–20].

In this work, we have successfully produced a highly aligned melt spun PCL fiber scaffold that allows fibroblast and VSMC aligned attachment and also preserves the contractile *α*-smooth muscle actin (*α*-SMA) expressing phenotype of the VSMC. This technique was also shown to be applicable for cell alignment on a prototype synthetic PLC vascular conduit.

## 2. Materials and Methods

### 2.1. Production of Melt Spun PCL

**Figure 1:**
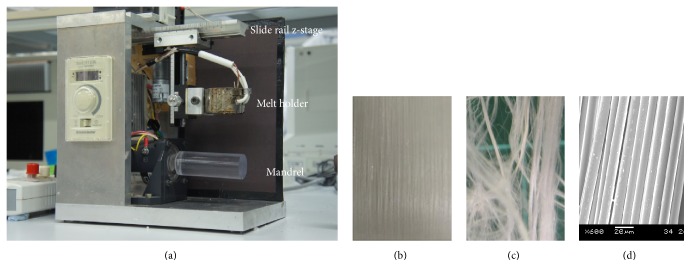
Melt spinning apparatus depositing fiber on a turning mandrel, demonstrating the slide rail, z-stage, melt holder, and mandrel; for more details see An et al. [21] (a). SEM image of the PCL fiber removed from the mandrel (b). PCL fibers without adhesive to maintain alignment (c), PCL fibers adhered into aligned orientation, using chitosan solution as an adhesive (d).

Melt spinning of polycaprolactone was performed as described in An et al. [21], using a customized apparatus as shown in Figure 1(a). In brief, powdered PCL (50 kDa) was melted at approximately 120°C and then was drawn by gravity to mandrel at 750 rpm [21].

The fibers were adhered into place by dip coating in 5% chitosan (w/v) in acetic acid and then incubated for 5 days at room temperature to allow solvent evaporation. As a control, chitosan films were formed by coating the base of 6-well plates with 500 *μ*L of 5% (w/v) chitosan in acetic acid followed by incubation for 5 days at room temperature in a fume hood to allow solvent evaporation.

### 2.2. A Dip Coated Prototype PLC Vascular Prosthesis

A PLC vascular graft was formed by dip coating a 5 mm diameter stainless steel mandrel in a 12% (w/v) PLC solution dissolved on chloroform using a Nima DC-Mono 300 dip coating apparatus. The PLC coating was incubated at room temperature for 48 hours to allow solvent evaporation. Subsequently the mandrel was placed into the melt spinning apparatus and a layer of circumferentially aligned PCL fibers was applied. The fibers were adhered using 5% chitosan solution as an adhesive as described above; following solvent evaporation the scaffold was coated in 1% gelatin to enhance cell attachment on the tubular surface.

### 2.3. Fourier Transform Infrared Spectroscopy

FTIR analysis was used to qualitatively characterize the functional groups introduced presented on the surface of the construct. FTIR spectra were collected with Frontier FT-IR spectrometer (PerkinElmer) at resolution of 4 cm^−1^ and signal average of 16 scans in each interferogram over the range of 4000–600 cm^−1^. Two measurements were retrieved on two random locations per sample for each group. Results were analyzed by plotting % transmission against wavelength (nm).

### 2.4. Atomic Force Microscopy (AFM)

Intermittent contact mode atomic force micrographs were obtained on JPK Instruments Nanowizard III (Aufgang C, Germany) with a nanoprobe of 100 *μ*m length and a rotated monolithic silicon narrow cantilever with a force constant of 40 Nm^−1^ and a tapping frequency of 300 KHz. The tapping mode AFM conditions were as follows: scan rate was 0.4 Hz, set point was 1.5 V, drive amplitude was set to 0.5 V, and the drive frequency was approximately 280 KHz. Adjustments of the integral gain, proportional gain, and set point were done to minimize contact force and electronic noise as well as to maximize the features of the sample. The AFM micrographs were evaluated by flattening the images (second-order) with the help of Nanowizard Data Processing JPK Software.

### 2.5. Cell-Seeded PCL Fiber Film

Smooth muscle cells (SMCs, Lonza) or human fibroblasts were cultured up to the 8th passage in smooth muscle cell basal medium (SmGM-2 Media (Lonza Bioscience)) or FibroGRO Complete Media (Millipore), respectively. PCL fiber films (1 × 1 cm^3^) were sterilized with 70% ethanol for 1 h and then were washed away with PBS (three times). The cells were seeded on the PCL fiber films and a control surface at a density of 5 × 10^4^ cells/cm^2^. Cell-seeded fibers were cultured in a flat bottom 24-well plate for 7 days.

### 2.6. Cell Seeding Prototype PLC Vascular Prosthesis

A 5 cm section of either uncoated or aligned PCL fiber coated conduits was placed in a 6 cm diameter dish. Subsequently, 3 mLs of a concentrated SMC suspension (5 × 10^6^ cells/mL) was slowly applied to the upper surface; after 10 min incubation at room temperature the tube was turned over and another 3 mLs of the cell suspension was applied. The constructs were incubated over night at 37°C in 5% CO_2_; then the cells morphology was examined using fluorescence microscopy.

### 2.7. Immunofluorescence Imaging with Confocal Microscopy

The seeded fibroblasts were recorded after 14 days. The fluorescence was generated by 30-minute incubation with 2 *μ*M calcein AM in PBS.

SMCs in cell-seeded fibers at day 3 and day 7 were fixed in 4% paraformaldehyde for 30 min in room temperature. Following fixation, fibers were washed 3x with PBS, permeabilized with 0.1% Triton X-100, and blocked using 2% BSA in PBS for 1 h at 4°C. After washing 3x at room temperature in PBS and immunohistochemistry labeling on fibers and control (chitosan film) samples was performed, applying primary antibody against alpha-smooth muscle actin (monoclonal mouse anti-human), at 1 : 100 dilutions in PBS/BSA/buffer at room temperature for 2 h. After washing 3 times for 10 min in PBS/BSA buffer, secondary antibodies (AF 488 goat anti-mouse, Invitrogen) were applied at 1 : 200 dilutions in PBS/BSA buffer at room temperature for 1 h. Cell nuclei were stained with DAPI (4′,6-diamidino-2-phenylindole) and PCL fibers were then imaged via confocal microscopy (Leica, Wetzlar, Germany).

## 3. Results

### 3.1. Characterization of Aligned Melt Spun Fiber Mat

The melt spin method produced a thin layer of highly aligned PCL fibers of 10 *μ*m width on a mandrel within approximately 15 minutes, creating aligning fibers of 10 *μ*m diameter (Figures 1(b) and 1(c)). The PCL is melt spun into nonbonded, distinct fibers that readily unravels (Figure 1(c)). Hence, the chitosan dip coating allows for an adhesive effect for the fibers to be removed from the mandrel as an aligned fiber mat (Figure 1(d)).

**Figure 2:**
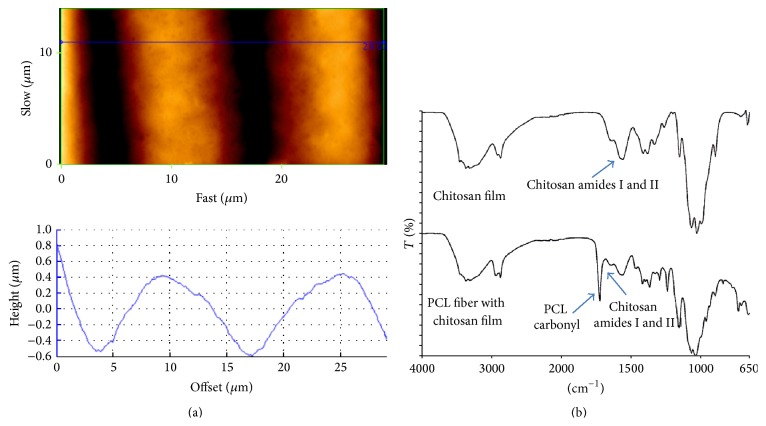
Surface analysis of the aligned chitosan covered fibers: AFM of fibers bonded by chitosan (a). FTIR of the chitosan film and PCL coated chitosan (b).

**Figure 3:**
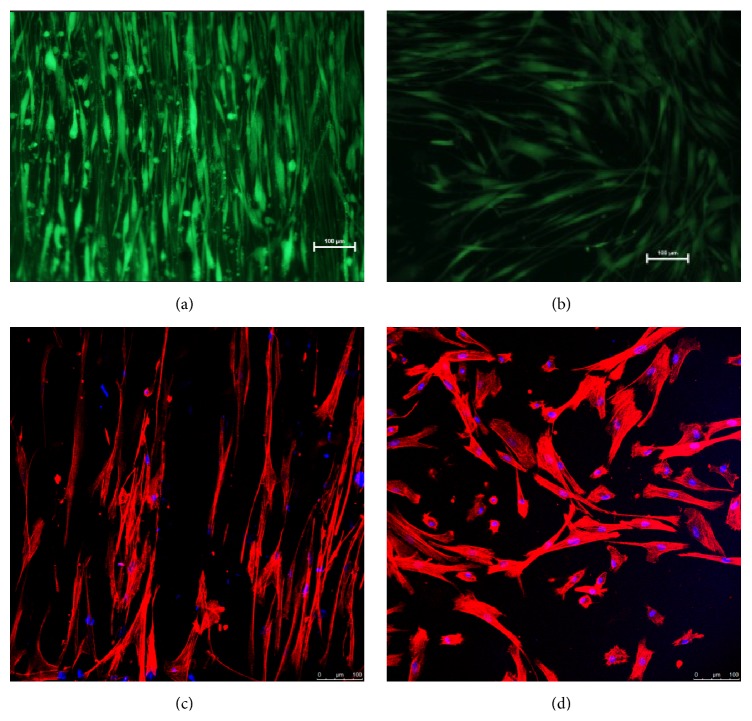
Fibroblasts cultures on aligned fiber (a) and on tissue culture plastic (b) for 14 days; cells are stained with calcein AM. SMCs orientated on melt spun aligned fiber (c) and cultured on chitosan film (d) for several days. The cells are immunostained for F-actin and DAPI nuclear stain.

The fiber mat was examined by AFM (Figure 2(a)) and FTIR (Figure 2(b)). The FTIR demonstrated a strong signal for the characteristic amide groups chitosan; hence the chitosan coats the fibers and becomes the principle substance cells will then interact with. The chitosan amide groups are known to help provide for a cytocompatible surface to the device. From the AFM, it appears the fibers form curved grooves approximately 10 *μ*m and 10 *μ*m deep, a depth which allows cells to make contact with cells growing in neighboring grooves as seen in Figures 3(a) and 3(c). In addition, the chitosan gives the scaffold a roughened surface feature, unlike the relative smooth surface of pristine PCL [22].

### 3.2. Aligned Orientation of Fibroblasts and Smooth Muscle Cells

Fibroblasts were seeded onto the fiber surface and readily aligned with the parallel fibers and became noticeably elongated compared to the control (Figures 3(a) and 3(b)). These cells continued to proliferate until reaching 100% confluency, producing a continuous, aligned monolayer (not shown); hence the fibroblasts were able to sense each other across channels. Similarly for SMCs, it was observed that after 7 days the cells adopted an elongated morphology following the orientation of the fiber, resembling the spindle-like appearance of the contractile cells (Figure 3(c)). In comparison the SMCs seeded on chitosan film grew without obvious orientation and present a rhomboid appearance akin to the secretory phenotype (Figure 3(d)). Interestingly, unlike the fibroblasts, the fiber seeded SMCs were not observed to proliferate towards confluency.

### 3.3. Expression of *α*-Smooth Muscle Actin (*α*-SMA)

**Figure 4:**
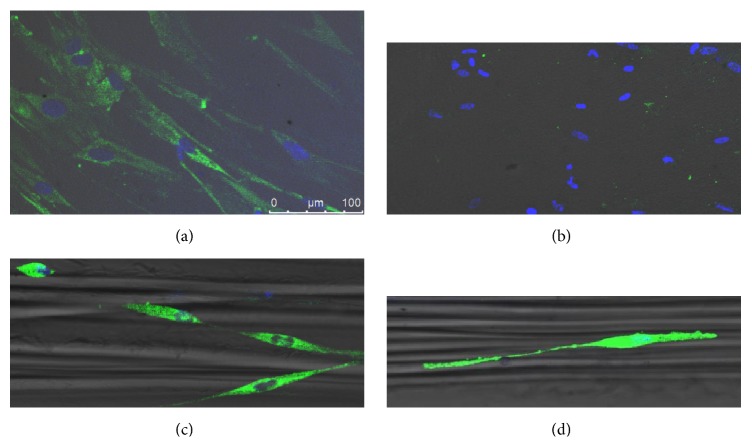
SMC expression of *α*-SMA. SMCs cultured on chitosan films for 3 days (a) and 7 days (b), SMCs on aligned chitosan coated PCL after 3 days (c) and 7 days (d). All cells stained for *α*-SMA and with DAPI nuclear stain.

The recently passaged smooth muscle cells (up to day 3) demonstrated positive staining by immunochemistry for the contractile phenotype marker protein *α*-SMA on both aligned fiber and nonaligning surface (Figures 4(a) and 4(c)). However, after several days the protein was only detectable in cells on the aligned fiber surface. This observation indicates that the fiber induced both cellular orientation and prolonged *α*-SMA expression and thus promoted the retention of the contractile phenotype (Figures 4(b) and 4(d)).

### 3.4. Application of Technology to a PLC Vascular Conduit

**Figure 5:**
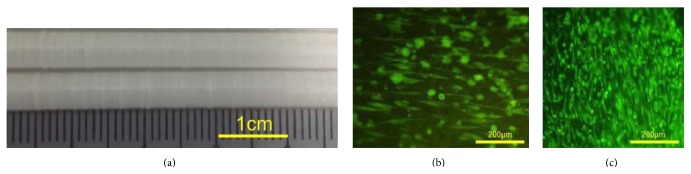
SMC seeding of a PLC prosthesis. A PLC tube was coated with PCL melt spun fiber (a), SMCs were seeded at high concentration (5 × 10^6^ cells/mL) on fiberless (b) and fiber coated (c) tubes, and images were recorded 5 days after seeding.

The melt spun fibers could be readily used to pattern the surface of a prototype PLC vascular conduit (Figure 5(a)). It was observed 48 hours after seeding the fiber covered and fiberless PLC tubes that VSMCs aligned longitudinally (Figure 5(b)) on the fiberless construct whereas, on the fiber decorated tubes, the VSMC had orientated along the fibers, giving a circumferentially aligned, elongated morphology. This pattern was still observed 5 days after seeding (Figure 5(c)).

## 4. Discussion

PCL based conduits are seen to have potential for the future fabrication of cellularized vascular prosthesis, with some successful clinical implantation [23]. SMCs are critical for the proper functioning of a blood vessel; however the synthetic phenotype is often associated with inflammation and graft failure. Hence the functional contractile phenotype should be promoted in a TEBV. Few attempts have been made to control SMC behavior on a cell-seeded tube [24, 25]. Here we test techniques of surface guidance, known to influence cell behavior on 2D surfaces, for their circumferential application around a conduit tube as required on a PCL vascular prosthesis. We are able to create cell aligning grooves using a method of melt spinning PCL which successfully aligned the orientation of both fibroblasts and SMCs and influenced the phenotype of SMC. Furthermore, we have demonstrated that polymer tubing (fabricated from PCL) on a mandrel can be decorated with circumferentially aligned melt spun PCL.

Fibers removed from the mandrel without the chitosan or gelatin bonding separated very readily. Furthermore polycaprolactone without modification has poor cell adhering properties, whereas chitosan with high deacetylation (85–95% in this case) has very good cell recruiting qualities. The higher the deacetylation, the greater the amount of free cationic amino groups that encourage cell adhesion to the surface [26, 27]. The smooth pristine PCL also lacks the surface roughness that usually facilitates the establishment of cellular adhesion points, whereas the chitosan coating gives a considerably more roughened surface feature. Furthermore, the AFM revealed that the chitosan covering does not fill in the space between fibers but instead leaves a distinct, curved groove that facilitates the aligned orientation of the cells.

Previous studies have demonstrated that SMCs are often seeded in a strongly contractile phenotype; then following a prolonged period of culture, the cells gradually become predominantly synthetic [7, 28]. Similarly in the present study we found SMCs seeded on chitosan films also began with notable expression of the contractile marker *α*-SMA. This expression is considerably diminished after several days, implying a move towards the secretory. The cells seeded on the aligned fibers also showed substantial expression of *α*-SMA 24 hours after seeding, and this expression was retained after several days, indicating the preservation of the contractile state [28, 29].

The contractile phenotype in this study was assessed by the elongated cell morphology and *α*-SMA expression; the latter is an important marker due to its dramatic loss during phenotype change during in vitro culture. There are several other key characteristics and marker genes that determine the SMC contractile phenotype. Chang et al. demonstrated that SMCs seeded on and elongating along similar micropatterned grooves expressed a greater number of contractile related genes and less proliferative related ones than SMCs grown on flat surface [30], thus proving the grooves promote the retention of the contractile phenotype.

The intracellular mechanism by which the contractile phenotype can be promoted in SMC elongating with cell aligning channels has been examined. There is a milieu of signal transduction pathways affecting SMC phenotype (as reviewed by [31]). However studies have been carried out on the mechanism of phenotype determination by surface features. Chang et al. detected strong activation of ERK and FAK, downstream signaling molecules from integrins, the surface adhesion proteins which “sense” the surface features. Thakar et al. [32] examined the effect of SMC shape on proliferation. They found a significant decrease in the expression of both the mRNA and protein of the nuclear receptor NOR-1 by elongated SMCs. NOR-1 appears to be associated with the characteristic proliferation of synthetic SMC. The knockdown of NOR-1 reduces SMC rate of proliferation [32].

Fibroblasts were able to align and become confluent on the fibers whereas SMCs differentiating towards contractile phenotype have a greatly reduced rate of proliferation [7, 8, 28]. To achieve confluency on the construct we expect the requirement to seed SMCs at a high cell density, hence conferring immediate confluency.

Several groups have created aligning scaffolds by electrospinning polymers. For example, Xu et al. [15] electrospun PLLA-CL to create an aligned scaffold for cell “contact guidance.” However, the method presented here has substantial advantages over electrospinning, such as the absence of the whipping action characteristic of electrospinning which reduces exact fiber alignment, as compared to melt spinning [33]. To get a highly aligned structure by electrospinning a narrow rotating mandrel collector is used for fiber collection thus generating aligning surfaces of limited width and with multiple layers of piled fibers whereas our melt spinning method produces a scaffold of 10 cm length and 1-fiber thickness. However an important advantage of electrospinning over melt spinning is the delivery of cells within the fibers for immediate and simultaneous deposition onto the graft within cytocompatible polymers and solvents [34, 35]. This will become an important technique if it is found that the SMCs adopt a contractile phenotype following delivery within circumferentially aligned electrospun fibers.

Unlike most work on cell aligning fiber scaffolds, the current study employed micron-diameter scaffolds. It has been demonstrated that PCL can be melt spun into fibers with range of diameters from 10 to 200 *μ*m [33]. The melt spun fibers we fabricate here have a diameter of 10 *μ*m; these generate interfiber grooves 10 *μ*m across, which matches the width of an elongated SMC. In a comparable study, on microsized fibers produced by wet spinning of PLGA, it was found that as the diameter of fibers increases from 10 *μ*m to 242 *μ*m, the degree of orientation decreases. The larger-diameter fibers conferred cellular alignment similar to planar, featureless surfaces [36]. Hence the fibers we created by melt spinning are in the favorable width range for promoting cell alignment. In addition, the relatively low depth for the groove allows fibroblast and SMCs to grow closely together as seen in Figure 3, unlike other methods that produce much deeper channels or wider intervals which keep cells separated across channels.

The melt spun fibers can be used to circumferentially align the SMCs on a prototype PLC vascular prosthesis in an elongated contractile-like phenotype thus demonstrating the effectiveness of the technique to orientate the cell both on flat 2D film and also circumferentially on the outer surface of a tube.

This ability of fabricating and controlling alignment patterns on tissue engineering scaffolds will aid the production of a more physiologically relevant representation of natural tissue [4]. Moreover, since the melt spun technique is delivered to a mandrel, it can be adapted to introduce circumferentially aligned pattern on the outer surface of a synthetic polymer TEBV, when placed in the position of the rotating mandrel. This allows the alignment of cells that comprise the outer vasculature layers, that is, fibroblasts and SMCs.

We aim to eventually achieve the coating of fully functional, vasoresponsive SMCs for the structure. However, SMCs can exist at various points of differentiation between the two phenotypes. Through contact guidance features we have successfully promoted a more contractile-like phenotype. To achieve fully functional contractile SMCs, we expect to involve additional steps [37] including seeding at high density, optimized cell harvesting techniques (enzyme digestion rather than outgrowths), growth factors (such as TGF-*β*), serum optimization, SMC source, and stimulation with pulsatile force. It is unlikely that only one of these approaches in isolation can achieve the formation of a vasoresponsive SMC medial layer; hence combinations may be more effective [28, 38].

In summary, the combination of aligned melt spun micron-sized fibers with a polymeric “adhesive” (chitosan) is a very promising substrate that enables the retention of the desired contractile phenotype of smooth muscle cells. Such a construct may be advantageously applied onto tubular constructs for forming a biomimetic blood vessel that will potentially have superior compliance matching with the native vessel.
